# Emotional Biases and Recurrence in Major Depressive Disorder. Results of 2.5 Years Follow-Up of Drug-Free Cohort Vulnerable for Recurrence

**DOI:** 10.3389/fpsyt.2019.00145

**Published:** 2019-03-28

**Authors:** Henricus G. Ruhe, Roel J. T. Mocking, Caroline A. Figueroa, Paulien W. J. Seeverens, Nessa Ikani, Anna Tyborowska, Michael Browning, Janna N. Vrijsen, Catherine J. Harmer, Aart H. Schene

**Affiliations:** ^1^Department of Psychiatry, Radboud University Medical Centre, Nijmegen, Netherlands; ^2^Donders Institute for Brain, Cognition and Behavior, Radboud University, Nijmegen, Netherlands; ^3^Department of Psychiatry, Location Academic Medical Center, Amsterdam UMC, University of Amsterdam, Amsterdam, Netherlands; ^4^Department of Psychiatry, University of Oxford, Oxford, United Kingdom; ^5^ProPersona Mental Health Care, Depression Expertise Center, Nijmegen, Netherlands; ^6^Department of Psychology, Behavioural Science Institute, Radboud University, Nijmegen, Netherlands

**Keywords:** major depressive disorder, remission, relapse, recurrence, emotional bias, prediction

## Abstract

An interesting factor explaining recurrence risk in Major Depressive Disorder (MDD) may be neuropsychological functioning, i.e., processing of emotional stimuli/information. Negatively biased processing of emotional stimuli/information has been found in both acute and (inconclusively) remitted states of MDD, and may be causally related to recurrence of depression. We aimed to investigate self-referent, memory and interpretation biases in recurrently depressed patients in remission and relate these biases to recurrence. We included 69 remitted recurrent MDD-patients (rrMDD-patients), 35–65 years, with ≥2 episodes, voluntarily free of antidepressant maintenance therapy for at least 4 weeks. We tested self-referent biases with an emotional categorization task, bias in emotional memory by free recall of the emotion categorization task 15 min after completing it, and interpretation bias with a facial expression recognition task. We compared these participants with 43 never-depressed controls matched for age, sex and intelligence. We followed the rrMDD-patients for 2.5 years and assessed recurrent depressive episodes by structured interview. The rrMDD-patients showed biases toward emotionally negative stimuli, faster responses to negative self-relevant characteristics in the emotional categorization, better recognition of sad faces, worse recognition of neutral faces with more misclassifications as angry or disgusting faces and less misclassifications as neutral faces (0.001 < *p* < 0.05). Of these, the number of misclassifications as angry and the overall performance in the emotional memory task were significantly associated with the time to recurrence (*p* ≤ 0.04), independent of residual symptoms and number of previous episodes. In a support vector machine data-driven model, prediction of recurrence-status could best be achieved (relative to observed recurrence-rate) with demographic and childhood adversity parameters (accuracy 78.1%; 1-sided *p* = 0.002); neuropsychological tests could not improve this prediction. Our data suggests a persisting (mood-incongruent) emotional bias when patients with recurrent depression are in remission. Moreover, these persisting biases might be mechanistically important for recurrence and prevention thereof.

## Introduction

Due to its high incidence, recurrence-rates and severity, Major Depressive Disorder (MDD) is a psychiatric disease which globally accounts for the greatest loss of years due to disability (1, 2). Defining predictors of recurrence that are preventable might help reduce this burden. The number of previous episodes of MDD is a strong predictor of both relapse and recurrence (1, 3, 4) —both are referred to as “recurrence” hereafter (5)– other predictors include the persistence of depressive symptoms (3) and coping style and/or daily hassles (1). However, in prior research, these predictors explained only ~29% of variance of time to recurrence (6)(ten Doesschate, Bockting, Koeter, Schene, & DELTA Study Group, 2010).

An interesting additional factor explaining recurrence risk in MDD may be neuropsychological functioning, especially the processing of emotional stimuli/information. Negatively biased processing of emotional stimuli/information has been found in different cognitive domains, both in acute and remitted states. An example of negative biases in emotional processing is *attentional* biases for negative stimuli, which have also been repeatedly observed in acute MDD patients (7–9). This bias consists of selective attention for negative stimuli, such as sad faces (10). Altered emotional processing is interpreted as a failure to suppress attention for negative stimuli (11–13). Moreover, MDD patients lack a positive attentional bias that is normally observed in healthy individuals, and show a decreased response to pleasant stimuli (14, 15). Interestingly, this type of altered emotional processing is suggested to have clinical correlates: it is associated with an impeded recovery from depression (10, 16, 17). Based on such findings, biased processing of emotional information is currently regarded as an important contributor to the onset of depression, and may therefore also be causally related to recurrence of depression (13, 18–22). Moreover, in the acute stage, MDD patients have difficulties in retaining positive or neutral information to their working memory and in blocking and removing negative information from working memory (23–25).

While abundant evidence shows that acutely depressed individuals differ in emotional processing from non-depressed controls, referred to as mood-congruent biases, little is known about how individuals with recurrent MDD in remission differ from those controls (26–28). Analogous, biases when in remission of depression could be considered mood-incongruent. Some of the neuropsychological deficits seen during an acute episode of MDD seem to persist between episodes, and the level of neuropsychological impairment might even be related to the number of previous depressive episodes (29). This may indicate that, as opposed to representing a state, (i.e., characteristics are only seen during a depressive episode), altered emotional processing represents a trait in individuals with increased risk of developing a first or recurrent depressive episode. This is further substantiated by (1) the presence of negative bias in never-depressed relatives of depressed individuals (who are at high risk for MDD), for example children of depressed mothers (30–32); (2) the relationship between bias and symptomatic improvement over time (10); and (3) the association of negative bias with depression candidate genes (33, 34). Also, a negative information processing bias was observed in highly neurotic but never depressed individuals (35). Negative biases in emotional processing might result in more frequent dysphoric states, leading to emotional vulnerability under stress and ultimately to depressive feelings (36, 37).

Indeed, alterations in emotional processing have been observed in MDD patients in remission compared to healthy (never depressed) controls, although this evidence is limited (38). For example, the negative attentional bias observed in depressed persons may in a lesser form persist or be reactivated during a sad mood in remitted depressed individuals; although results are mixed (19, 39–45). Other biases in remitted patients concern the *negative interpretation* of neutral or ambiguous information (46), preferential recall of negative material (46–50), a *reduced error monitoring* due to prolonged emotional disturbance after self-monitored errors, decreased *learning* and a *ruminative thinking style* when confronted with negative information (13, 20). Moreover, cognitive effects seem to be greatest when emotional stimuli match the domain of greatest concern to the subject, e.g., represent self-referential information (51). However, the differences above have often only been observed when remitted depressed individuals are in a dysphoric mood or stressed, suggesting that these biases are activated by decreased mood (i.e., mood-congruent). In sum, evidence for the persistence of alterations in emotional processing (as a trait) during remission is not conclusive yet.

Importantly, the presence of neuropsychological differences between individuals vulnerable for recurrence of MDD and never-depressed controls does not necessarily imply that these neuropsychological processes are predictive of future recurrence. Until now only one study investigated the relation between self-referential emotional biases and recurrence (49). If such an association is replicated, alterations in emotional processing could be implicated to predict or recognize a preceding new episode at an early stage. Moreover, interventions to modify emotional processing biases have been developed for depressed individuals (37, 52–59). Therefore, if a relation between emotional biases and recurrence exists, this type of bias-modification intervention could also have a preventive effect in MDD patients in remission.

We therefore aimed to investigate self-referent, memory and interpretation biases in recurrently depressed patients in remission. To avoid any influence of medication on emotional processing (60), we only included participants who were voluntarily free of antidepressant maintenance therapy for at least 4 weeks. For the exploration of the different cognitive domains of altered emotional processing, we used tasks specifically designed to disentangle these. First, to test bias in self-referent information processing, we used an emotional categorization task (61). We presented positive and negative words describing a characteristic, and asked the subjects if they would appreciate this trait as desirable or undesirable. We hypothesized that subjects remitted from MDD would need less time to process negative trait words than controls and that shorter processing time would be associated with recurrence. Second, to explore possible bias in emotional memory we subjected the remitted depressed individuals to a free recall of the emotion categorization task stimuli exactly 15 min after completing it (61). We hypothesized that subjects remitted from MDD would (1) remember more negative words than controls and (2) show more negative memory intrusions compared to never-depressed controls. We also expected the strength of the negative bias to be associated with recurrence and time to recurrence. Third, we used a facial expression recognition task (61), to test if alterations in recognition and reaction times would occur to faces with negative and positive expressions. We hypothesized that remitted MDD subjects would show (1) faster and better recognition of negative expressions compared to controls, (2) a slower recognition of positive expressions compared to controls, and (3) as in the previous task, that these effects would be associated with recurrence.

## Materials and Methods

### Participants

We recruited patients with recurrent MDD currently in remission [≤7 for ≥8 weeks on the Hamilton depression rating scale (HDRS) (62)] and not fulfilling the criteria for a current MDD episode–as assessed using the structured clinical interview for DSM-IV disorders [SCID-I (63) during inclusion]; between 35 and 65 years, with 2 or more MDD episodes according to the SCID-I. All participants gave written informed consent. The study was approved by the local Medical Ethics Committee of the Academic Medical Centre, Amsterdam, The Netherlands.

As described in our methods-paper (64), we recruited participants (and controls) via advertisements and via databases registering previous clinical treatment and/or participation in previous studies at the mood disorder department. In addition, we contacted patients with a known recurrent MDD without current medication through their general practitioners who have an affiliation with the Academic Medical Centre of Amsterdam (AMC).

Participants did not take psychopharmacologic drugs for at least 4 weeks, although we allowed incidental benzodiazepine use, as long as this could be stopped after informed consent. Exclusion criteria were current diagnosis of alcohol or drug dependence, psychotic or bipolar disorder, predominant anxiety disorder, electroconvulsive therapy within 2 months before assessment or a history of head trauma or neurological disease or severe general physical illness.

We likewise recruited never-depressed controls, free of lifetime psychopathology, throughout the study, who were matched on age (±3 years), sex and estimated intelligence (Dutch adult reading test (DART (65)); with a HDRS ≤ 7. Exclusion criteria for controls (as far as applicable) were identical to MDD-patients.

### Clinical Assessment

After informed consent, we administered the SCID-I (63) to ascertain current and past depressive episodes, HDRS and IDS-SR (63) by phone interview, to ensure that participants did not meet criteria for a depressive episode, and—for the MDD group—were in remission. We thereafter scheduled a visit to our lab and requested participants to abstain from caffeinated drinks before performance of the tasks.

After instruction of the tasks and anthropomorphic measures, participants performed neuropsychological tasks in 2 blocks separated by a break. For description of the full baseline assessment see Mocking et al. (64).

### Cognitive Tasks

#### Emotional Categorization (EmCAT)

In this task, 60 personality characteristics selected to be disagreeable or agreeable (i.e., valence) were presented on the computer screen for 500 ms each. The task lasted for 6 min [for a complete description of the task, see (66)]. Characteristics were translated from the original English version to Dutch (and back-translated), matched in terms of word length, ratings of usage frequency, and meaningfulness. Participants were asked to categorize the words as likable or dislikeable as quickly and accurately as possible. Specifically, they were asked to imagine whether they would be pleased or upset if they overheard someone else referring to them as possessing this characteristic, so that the judgment is self-relevant and in part (but deliberately less explicitly) self-referent than e.g., the self-referential encoding task (SRET) (67, 68). The emotional categorization task was followed by administration of the DART and a short break.

#### Emotional Memory Task (EmMem)

Exactly 15 min after completion of the emotional categorization task, participants were asked to recall as many personality characteristics as possible. The number of positive and negative words recalled was computed for correct and false responses. The aim of this task was to test if participants with recurrent MDD recalled more negative words and had more negative intrusions (recalling words that were not in the EmCAT) than the healthy control group.

#### Facial Expression Recognition Task (FERT)

Six basic emotions (happiness, surprise, sadness, fear, anger, disgust) from 10 different individuals from the Pictures of Facial Affect series (69), were morphed between each valence and neutral and presented in a random order for 500 ms, followed by a blank screen. Participants were instructed to respond as quickly as possible and indicate the emotion they recognized by pressing one of six designated keys on the keyboard. This task lasted for 20 min and has been extensively validated before (60, 61).

### Follow-Up

We performed a follow-up of the recurrent MDD-participants by regular (every ~4 months) phone-calls, during which the SCID and HDRS were administered (64). To maximize the detection rate of recurrences, we also instructed participants to contact us when they subjectively experienced a recurrence and informed a person close to them about these instructions.

### Statistics

We used IBM's SPSS version 25.0 (SPSS Inc., Chicago IL, USA); we considered *p* < 0.05 as threshold for statistical significance. With power = 0.80 and two-tailed α = 0.05, our sample size of 69 MDD-patients and 43 controls allowed us to detect effects with a small effect size for ANOVA-based repeated measures analyses (>0.13) and moderate effect-sizes (>0.55) with independent *t*-tests (G^*^Power 3.1.9 Kiel, Germany). In case a patient or control did not complete a cognitive task, the subject was excluded for the analyses of that task. The computerized tasks prevented the occurrence of missing reaction times or accuracy when a task was completed.

#### Comparisons Between rrMDD-Patients and Controls

First, we calculated means for demographic and clinical variables. We assessed normality and compared baseline characteristics between patients and controls using independent samples *t*-test, χ^2^ tests or Mann-Whitney U test for non-parametric data, as appropriate.

For the EmCAT, we first checked occurrence of outliers and extreme reaction times, and then calculated the mean accurate classifications and reaction times per subject. We first compared reaction times for accurate and inaccurate categorization of positive/negative characteristics using independent *T*-tests. For accurate responses, we investigate effects of valence and valence^*^group interactions with a repeated measures ANOVA. Finally, to investigate combined contrasts of positive/negative characteristics, accuracy and group (i.e., valence^*^group^*^accuracy interaction), we applied linear mixed models with group as a between-subject factor (patients, controls), emotional valence as a within-subject factor (negative characteristics vs. positive), accuracy (correct/incorrect) and reaction time as dependent variable.

For the EmMem a 2 × 2 × 2 repeated measures ANOVA was calculated, with group (patient, controls) as the between-subject factor and false vs. correct answers and positive vs. negative words as within-subject factors.

For the FERT we compared reaction times and (mis-)classifications between groups per valence with independent sample *t*-tests and the interactions of (mis-)classification^*^group with repeated measures ANOVA. We used a linear mixed model procedure with group as a between-subject factor (patient, controls) and emotional expression as a within-subject factor (angry, fearful, sad, disgusted, neutral, surprised and happy; grouped as negative, neutral and positive faces) with reaction time as the first outcome variable and accuracy as a secondly tested variable.

#### Associations With Recurrence

For associations with recurrence in remitted MDD-patients, in order to avoid circular associations and reduce the number of variables to be examined in association with recurrence risk, we used the significant differences and interactions with controls (previous section) to calculate outcome-specific composite scores (definitions provided in **Tables 4**, **5**). First, we compared baseline results of these outcomes for rrMDD-patients with and without a recurrence during prospective follow-up. Second, in order to take into account the time to the (depressive) event or censoring by loss to follow-up, we used Cox proportional hazards regression models, with time to first recurrence as primary outcome. Participants lost to follow-up or without relapse during follow-up were considered censored. Because the number of previous depressive episodes and residual depressive symptomatology have been established as independent predictors of recurrence (1, 3, 4), we included these variables in all models. As independent variables, we used the significant differences and interactions with controls (i.e., the outcome-specific composite scores). We used a forward stepwise inclusion of all independent variables for each task separately. Finally, we for each task, we developed a task-specific composite score by using all outcomes of a task in a logistic regression to predict whether a subject would be a rrMDD-patient or control. Of this prediction-model we saved the standardized residuals of each task per subject and used this as a composite score (i.e., representing the individual's deviation of the general model). These task-specific composite scores were then planned to be used in the Cox-models assessing the independent contributions of the emotional bias test-battery by (1) entering the three task-specific composite scores per task as separate predictors and (2) by entering the three task-based composite scores simultaneously.

#### Machine-Learning Approach to Predict Recurrence

Given the many outcome variables generated by the EmCAT, EmMem and FERT, the acknowledged multiple comparison problem when testing these in individual models and the risk of overfitting models with relatively few cases, we applied a data-driven machine-learning approach to investigate prediction of recurrence, irrespective of the patient-control comparison.

As described for predicting treatment-response by the same neurocognitive test-battery (70), a linear support vector machine (SVM) was used to combine demographic (extended with the Childhood Trauma Questionnaire (CTQ) (71, 72) questionnaire) and task features into binary predictions (i.e., recurrence/non-recurrence). SVMs are a widely used and robust method of deriving binary classifications, particularly when the ratio of data points to features is relatively low, like in this study. Analysis was performed using Matlab (version R2014b, Mathworks). Performance of the algorithm was assessed using a leave-one-out validation procedure during which a training set consisting of all but one participant was used. The training set was used for feature selection, estimation of the C- parameter and model training, with the left out sample being used solely for validation (73). Note that this approach results in variability in the features selected, the C-parameter used and the model weights for each iteration of the leave-one-out procedure. The value of the C-parameter used was selected based on the achieved accuracy within the training set using 50 values of the parameter ranging from 0.01 to 100. Feature selection was achieved by selecting the features with the highest area under the curve for predicting recurrence in the training set. Missing values of a given feature in either the training or testing set (e.g., reaction times for choices, which were not made by a particular participant could not be calculated) were entered as the mean value for that feature, calculated from the training set. The unbalanced nature of the data set (i.e., unequal numbers of recurrent and non-recurrent patients) was dealt with by setting the weight of each observation to 1/(number of observations of a given class) in the training set (74).

Separate analyses were completed to test the predictive ability of the emotional bias tasks, residual symptoms and previous episodes, extended with childhood adversity (CTQ). Selection of variables/task features was independent of previous analyses. We then used different proportions of task features (10, 50, or 100% of available features). The rationale for assessing this range of proportions of task features is that, if most information about recurrence is contained in only a few task features then the classifier which uses just these features will perform better, whereas if information about recurrence is distributed throughout many task features, then the more inclusive classifiers will perform better. Significance (*p* < 0.05) of the classifier was determined based on accuracy relative to the *a-priori* recurrence rate in this sample (54.7%). We calculated the z-score for difference between proportions, and considered one-sided *p*-values, given the expected better performance of the classifier.

## Results

**Figure 1:**
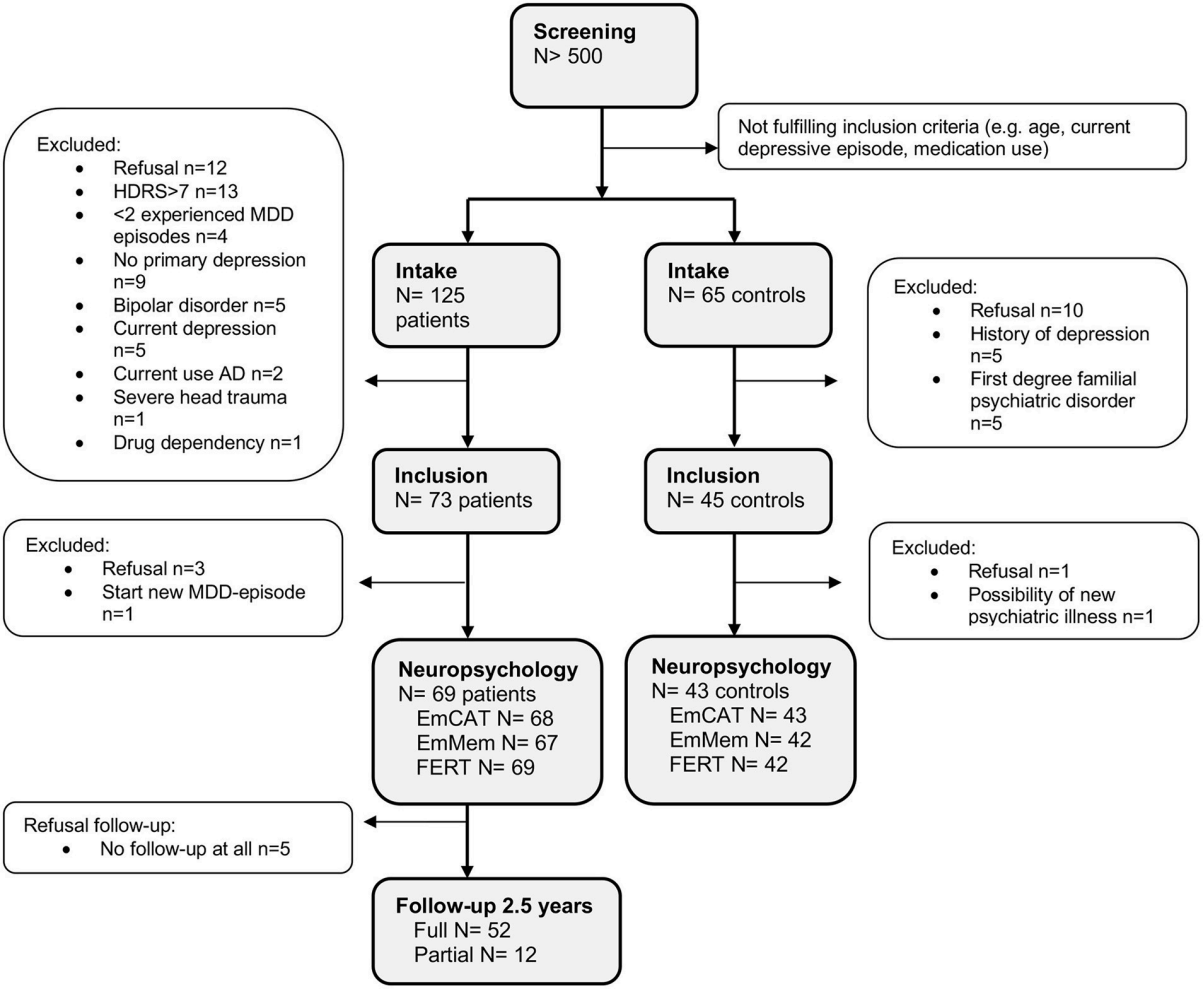
Disposition of participants. AD, Antidepressants; HDRS, Hamilton Depressive Rating Scale; MDD, Major Depressive Disorder.

We included 73 remitted MDD-patients and 45 controls. Of these, 69 MDD-patients and 43 controls completed the neuropsychological test battery. Of the 69 MDD-patients, 64 (92.8%) had at least 1 follow-up measurement and 52 (75.4%) completed follow-up for 2.5 years (Figure 1).

The groups did not differ significantly on age, gender, intelligence score, education (75) and living situation (all *p* > 0.05; Table 1). However, remitted MDD-patients were significantly less often employed compared to controls (*p* = 0.04) and had a slightly but significantly higher HDRS-score than controls (Mann-Whitney; *p* < 0.001).

**Table 1:** Remitted recurrent MDD patients vs. controls at baseline.

				**Between-group statistics**			
		**rrMDD (*n* = 69)**	**HC (*n* = 43)**	**χ^2^**	***T***	***U***	***p***
Female	*N* (%)	45 (65.8%)	30 (69.8%)	0.25			0.68
Age	Years; mean (SD)	53.4 (7.7)	51.5 (8.2)		1.20		0.23
Education	Levels^a^	0/0/0/4/22/27/16	0/0/0/1/16/18/8	1.25			0.76
IQ	Mean (SD)	108.8 (8.2)	106.3 (9.6)		1.43		0.16
Living situation	Levels^b^	29/0/19/17/2/0/2	12/0/16/11/4/0/0	5.52			0.24
Employment status	Levels^c^	26/27/16/0	21/17/5/0	2.68			0.26
Currently employed	Yes (%)	46 (68.7)	37 (86.0)	4.28			0.04
Age of onset	Years; mean (SD)	26.7 (10.8)	–				–
Episodes	Median (IQR)						
last 10 years lifetime		2 (1–2) 4 (2–7)	–				–
HDRS	Median (IQR)	2 (0–5)	1 (0–1)			2,317	0.001
Childhood adversity (CTQ)	Mean (SD)						
Total Emot. abuse Phys. abuse Sex. abuse Emot. neglect Phys. neglect		49.8 (14.4) 11.6 (5.5) 6.4 (2.8) 6.7 (3.1) 16.1 (5.3) 8.9 (3.4)	35.4 (12.7) 6.9 (3.5) 5.6 (1.7) 6.0 (3.0) 10.2 (4.3) 6.7 (3.0)		5.31 5.47 1.90 1.20 6.10 3.32		<0.001 <0.001 0.60 0.23 <0.001 0.001

*CTQ, Childhood Trauma Questionnaire; HC, Healthy Control; HDRS, Hamilton Depression Rating Scale; rrMDD, remitted recurrent major depressive disorder; LEIDS-R, Leiden Index Depression Sensitivity-Revised; RRS, Ruminative Response Scale*.

a *Level of educational attainment (70): primary school not finished/primary school finished/primary school + ≤ 2 years of lower level secondary school finished/lower level secondary school finished/medium level secondary school finished/high level secondary school finished/pre-university or university degree)*.

b *Living situation: alone/living with parents/cohabiting/cohabiting with children/single living with children/other/unknown*.

c *Employment status, low/middle/high/never worked; IQR, Inter-quartile range; χ^2^, chi-square test statistic; p, p-value; U, Mann-Whitney U non-parametric test statistic; T, independent-samples T-test statistic*.

### Baseline Measurements

#### EmCAT

We excluded 1 rrMDD-patient who did not complete the task. The EmCAT was performed correctly by most individuals: 35 of 68 rrMDD-patients and 22 of 43 controls had no inadequate responses to positively or negatively valenced characteristics. In direct groupwise-comparisons of reaction time for positive/negative characteristics and accuracy thereof, only for misclassifications of negative characteristics rrMDD-patients had longer reaction times relative to controls (Table 2). When we restricted analyses to accurate responses, both rrMDD-patients and controls showed faster reaction times for, and better recognition of positive characteristics (significant main effect of valence; repeated measures ANOVA; [*F*_(1,109)_ = 66.41; η^2^ = 0.38; *p* < 0.001] and [*F*(_1,109)_ = 27.84; η^2^ = 0.20; *p* < 0.001], respectively). There were no significant differences between rrMDD-patients and controls, nor was there a significant group^*^valence interaction (all *p*'s > 0.086). However, when we corrected for baseline differences in HDRS-scores between groups, for reaction times the group^*^valence interaction became significant [*F*_(1,107)_ = 4.85; η^2^ = 0.04; *p* = 0.03], with patients being faster in negative and slower in positive characteristics, as compared to controls.

**Table 2:** Baseline comparisons of emotional biases in rrMDD vs. controls.

				**Between-group statistics**			
		**rrMDD** **(*n* = 69)**	**HC** **(*n* = 43)**	***T***	***F* (df)**	***U***	***p***
**EMOTIONAL CATEGORIZATION (EmCat)**^§^							
RT Neg. Acc.	ms (SEM)	1,084.3 (28.6)	1,110.6 (39.3)	0.551			0.58
RT Neg. Mis.^*^	ms (SEM)	1,246.0 (60.6)	1,036.9 (68.8)	−2.249			0.03
RT Pos. Acc.	ms (SEM)	980.2 (30.6)	950.2 (29.2)	−0.668			0.51
RT Pos. Mis.^*^	ms (SEM)	1,351.4 (127.1)	1,082.0 (152.4)	−1.329			0.19
Count Neg. Acc.	median (range)	28 (14)	28 (13)			1,432.5	0.85
Count Neg. Mis.	median (range)	2 (14)	2 (13)			1,491.5	0.85
Count Pos. Acc.	median (range)	29 (12)	29 (5)			1,598	0.38
Count Pos. Mis.	median (range)	1 (12)	1 (5)			1,326	0.38
**EMOTIONAL MEMORY (EmMem)**^§^							
Count Neg. Acc.	median (range)	3.0 (7)	3.0 (10)			1,473.5	0.67
Count Pos. Acc.	median (range)	3.0 (7)	3.0 (10)			1,479.5	0.65
Count Pos. New	median (range)	2.0 (9)	2.0 (8)			1,151.0	0.11
Count Neg. New	median (range)	1.0 (7)	1.0 (4)			1,394.5	0.93
**FACIAL EMOTION RECOGNITION (FERT)**^§^							
RT Angry	ms (SEM)	2,053.3 (62.5)	2,108.5 (123.7)	0.441			0.66
RT Fear	ms (SEM)	2,241.5 (63.7)	2,208.0 (82.0)	−0.322			0.75
RT Sad	ms (SEM)	2,440.6 (71.9)	2,314.4 (119.2)	−0.906			0.37
RT Disgust	ms (SEM)	2,179.1 (68.9)	2,303.8 (111.0)	0.954			0.34
RT Neutral	ms (SEM)	1,788.2 (71.2)	1,643.5 (87.1)	−1.274			0.21
RT Surprise	ms (SEM)	2,251.5 (60.8)	2,210.7 (95.7)	−0.379			0.71
RT Happy	ms (SEM)	1,754.9 (38.1)	1,700.7 (62.6)	−0.786			0.43
RT Negative	ms (SEM)	2,225.8 (37.1)	2,230.3 (47.4)		0.005 (1,415)		0.94
RT Positive	ms (SEM)	1,871.9 (39.3)	1,820.8 (50.0)		0.654 (1,154)		0.42

* *As not all subjects misclassified characteristics during emotional categorization, these mean reaction-times are based on less subjects*.

§ *Due to missing tasks for the EmCAT n = 68 rrMDD and 43 controls, for the EmMem n = 67 rrMDD and 42 controls, and for the FERT n = 69 rrMDD and 42 controls*.

When we examined combined contrasts between positive/negative characteristics and groups in more sophisticated mixed models, regarding accuracy rrMDD made more mistakes (significant main effect of group; mixed model; [*F*_(1,4787.91)_ = 3.91; *p* < 0.048] and we observed more mistakes for negative characteristics (main effect for valence; [*F*_(1,4787.91)_ = 39.62; *p* < 0.001] without a group^*^valence interaction (*p* = 0.56). When we corrected for baseline differences in HDRS-scores between groups, significance of the difference between rrMDD and controls was lost (*p* = 0.13).

**Figure 2:**
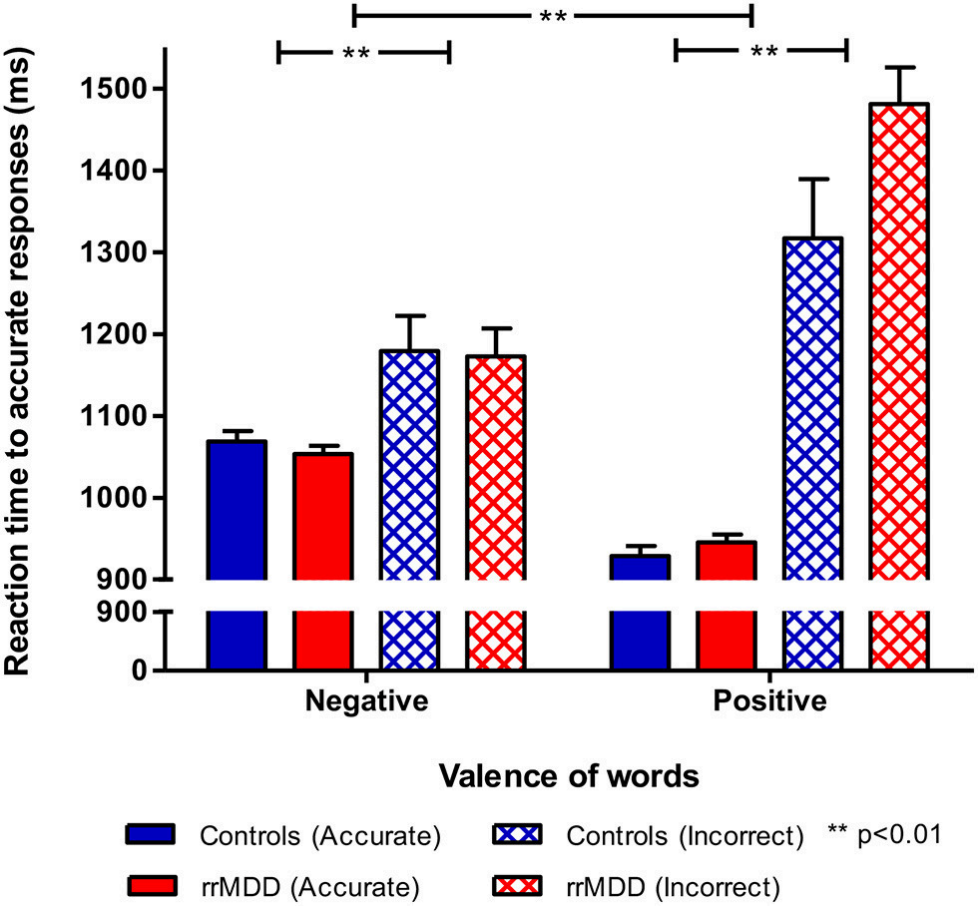
Reaction times by accuracy in rrMDD-patients and controls for the emotional categorization task. Figure shows reaction times of rrMDD patients vs. controls, distinguishing responses that are accurate positive/negative or inadequate. In a mixed model reaction times were slower for incorrect responses (main effect for accuracy; *p* < 0.001) and the accuracy*valence*group interaction was significant (*p* < 0.001). Relative to controls, rrMDD-patients were faster in response to negative characteristics and slower in response to positive characteristics, while especially for incorrect responses to characteristics words this difference was the largest. **p < 0.01; rrMDD, remitted recurrent depressive disorder.

For the reaction times, we examined the valence^*^accuracy^*^group interaction. Overall reaction times were longer for incorrect responses (main effect for accuracy; mixed model; [*F*_(1,4265.28)_ = 123.94; *p* < 0.001]). Moreover, the accuracy^*^valence^*^group interaction was significant [*F*_(2,3810.45)_ = 30.99; *p* < 0.001). Relative to controls, rrMDD-patients were faster in response to negative characteristics and slower in response to positive characteristics, while especially for incorrect responses to positive characteristics this difference was the largest (Figure 2). When correcting for baseline differences in HDRS-scores between groups, results were similar, except that an overall slower response to positive relative to negative characteristics became significant [*F*_(1,4254.32)_ = 4.48; *p* = 0.03] too.

#### EmMem

**Figure 3:**
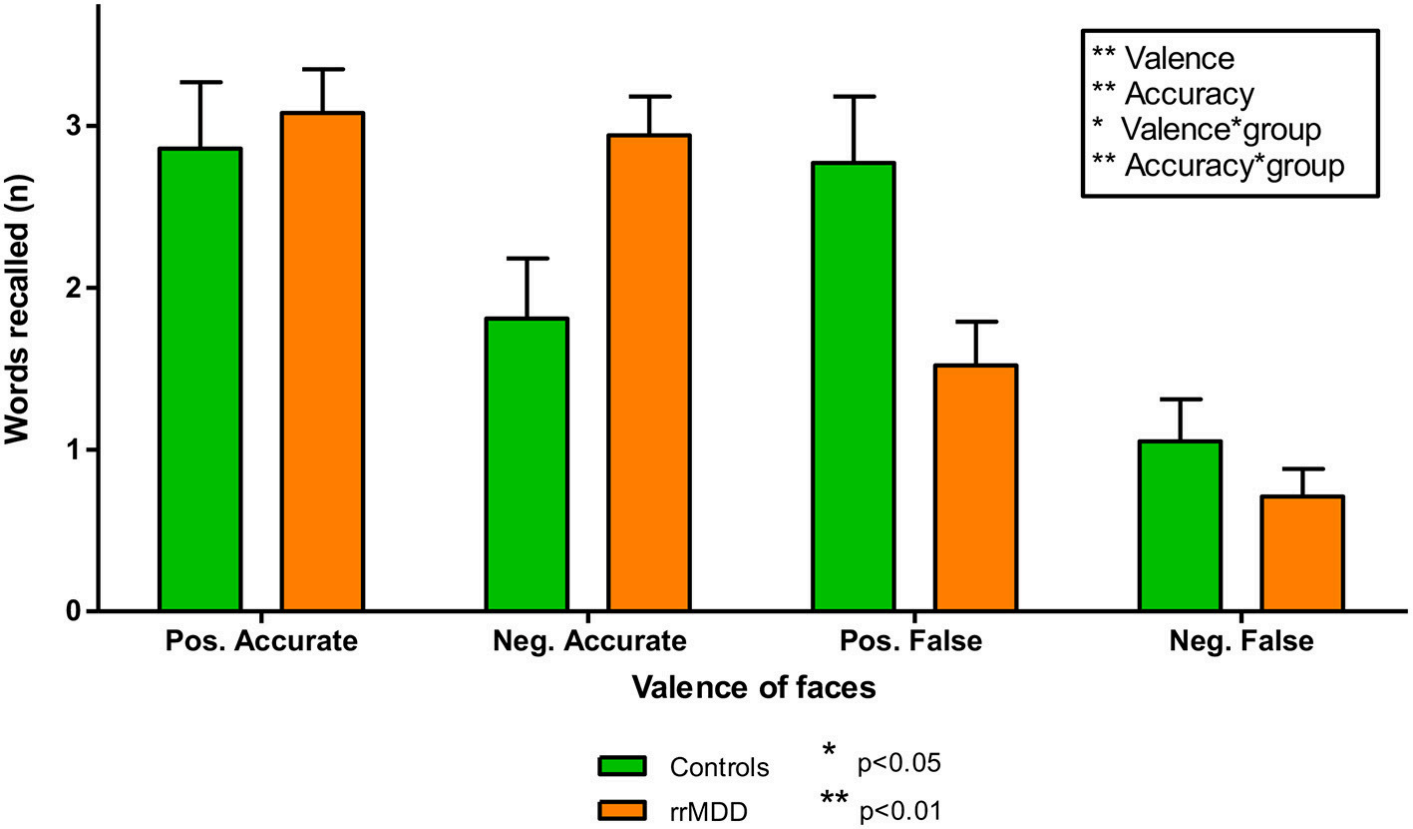
Accurately and falsely endorsed characteristics in rrMDD-patients and controls for the emotional memory task. Figure shows the number of characteristics reported by rrMDD patients vs. controls, distinguishing characteristics that are accurately or falsely endorsed. In a repeated measures ANOVA, we found significant main effects for valence (better recall of positive characteristics; *p* < 0.001) and accuracy (overall more characteristics were correctly endorsed; *p* < 0.001), with a significant accuracy*valence interaction (no difference between positive and negative characteristics when recalled correctly, but more positive than negative characteristics when recalled incorrect; *p* < 0.001). However, the accuracy*valence*group interaction was not significant (*p* = 0.24). *p < 0.05; **p < 0.01; rrMDD, remitted recurrent depressive disorder.

We excluded 2 rrMDD-patients and 1 control who did not complete the task. In direct comparisons of patients and controls regarding separate outcomes we found no significant differences (Table 2). We examined the accuracy^*^valence^*^group interaction in the recall of positive and negative characteristics with a repeated measures ANOVA, also taking into account that participants falsely remembered positive/negative characteristics (Figure 3). Both in rrMDD-patients and controls, we found a better recall of positive characteristics (main effect of valence; [*F*_(1,107)_ = 26.65; η^2^ = 0.20; *p* < 0.001]) and overall more characteristics were correctly remembered (main effect of accuracy; [*F*_(1,107)_ = 46.00; η^2^ = 0.30; *p* < 0.001]). In addition, we found a significant accuracy^*^valence interaction (no difference between positive and negative characteristics when recalled correctly, but more positive than negative characteristics when recalled incorrect; [*F*_(1,107)_ = 19.08; η^2^ = 0.15; *p* < 0.001]). However, there was no significant accuracy^*^valence^*^group interaction (*p* = 0.24). Correction for baseline HDRS differences between groups did not change these findings.

#### FERT

**Figure 4:**
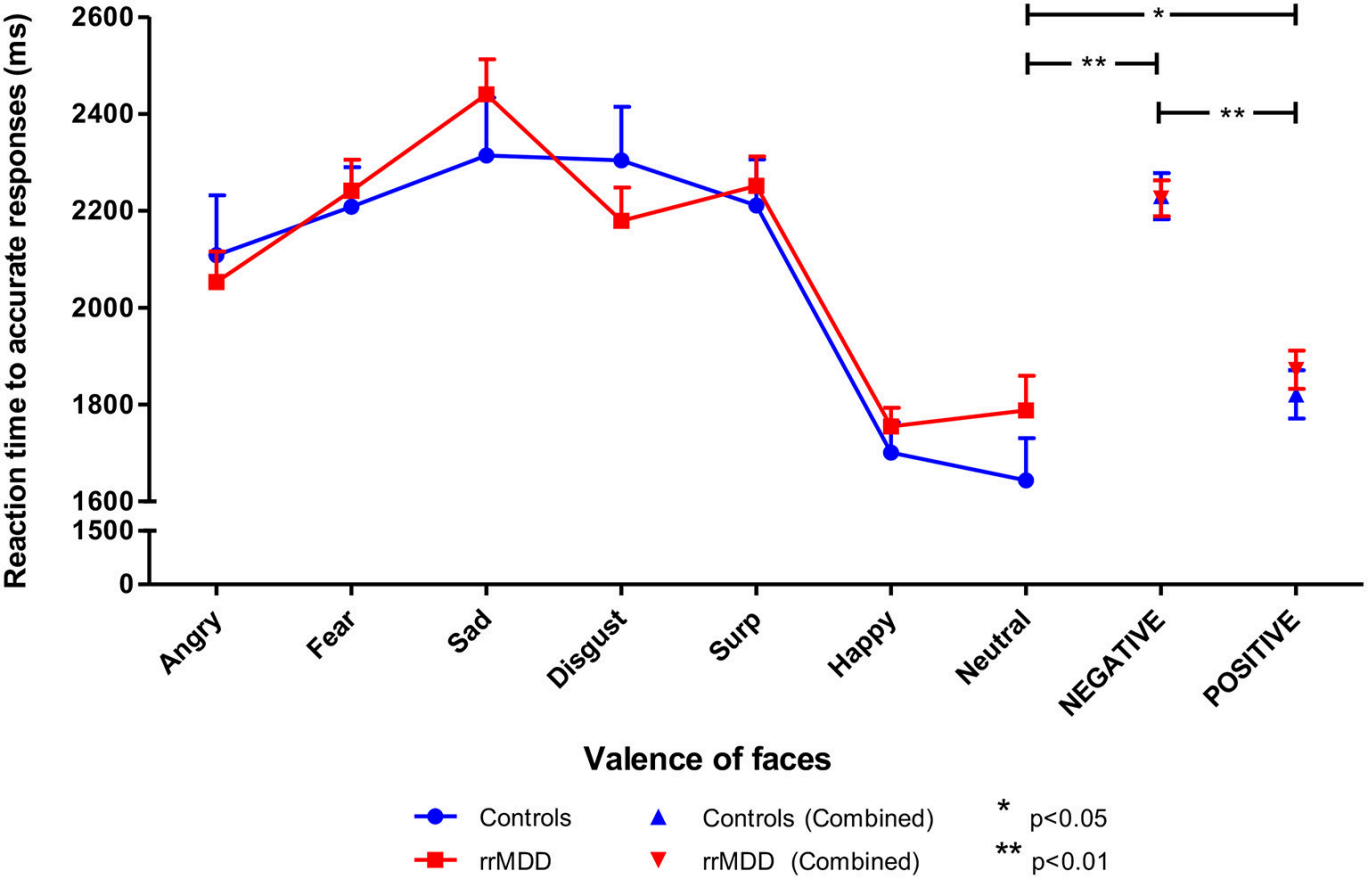
Reaction times in rrMDD-patients and controls when performing the facial expression recognition task. Figure shows the reaction times to emotional expressions (irrespective of accuracy of recognition) in rrMDD-patients and controls. At the right, the valences angry, fear, sad and disgust are combined as negative, while surprise and happy are combined as positive emotions. There were no differences in reaction-time between rrMDD-patients and controls for any emotion. There was a significant main effect of valence (*p* < 0.001), with significant slower reaction-times for negative (*p* < 0.001) and positive (*p* = 0.046), relative to neutral faces, but without a significant main group effect or valence*group interaction (*p* > 0.248).*p < 0.05; **p < 0.01; rrMDD, remitted recurrent depressive disorder.

**Figure 5:**
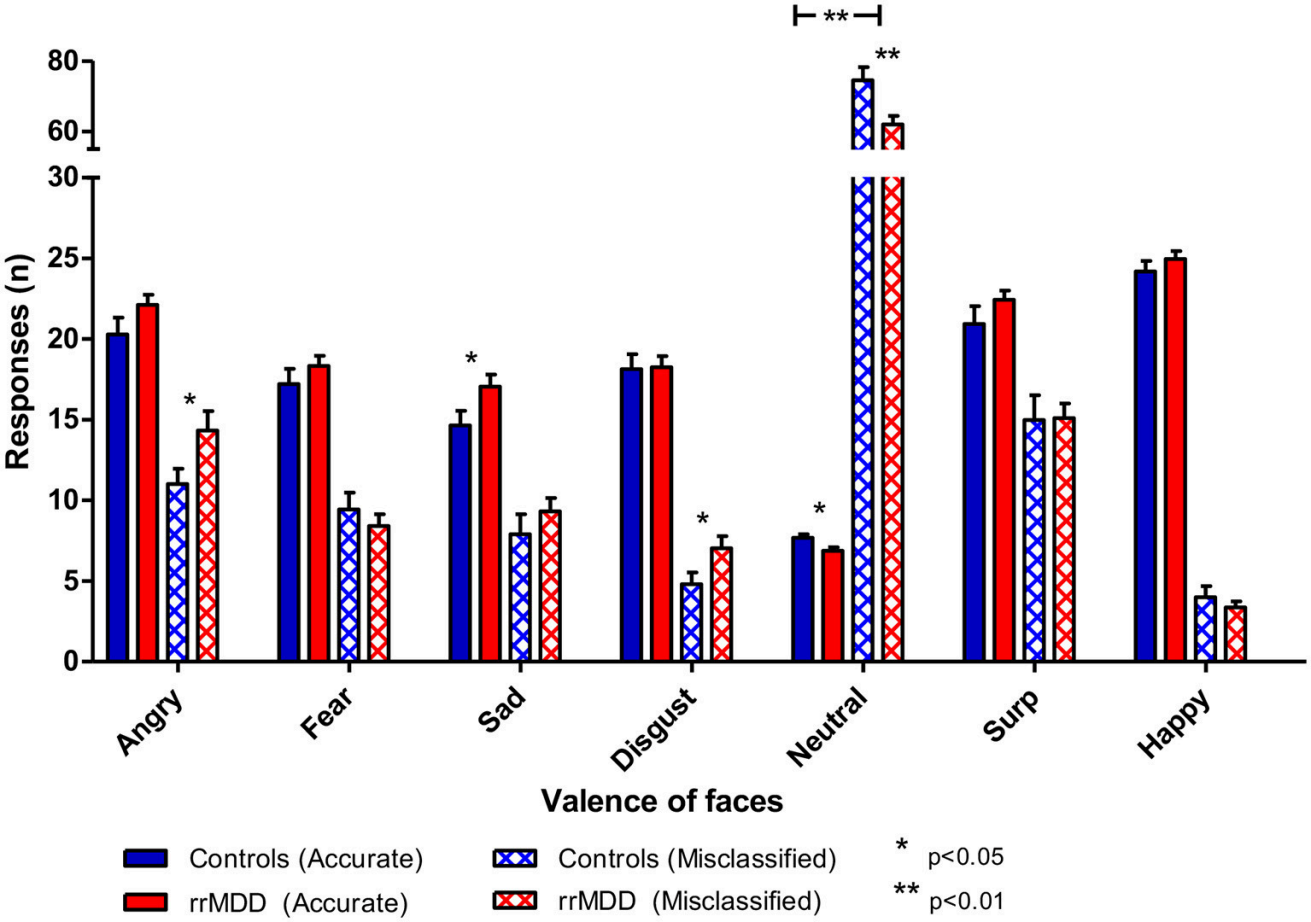
Accuracy of recognition of 7 valences of facial expressions by rrMDD-patients vs. controls for the facial expression recognition task. Figure shows the number of emotional expressions (7 valences) accurately recognized and misclassified as the indicated emotion for rrMDD-patients and controls. There was an increased recognition of sad faces (p=0.047), higher misclassification as angry (p=0.048) and disgusting (p=0.046), worse recognition of neutral faces (p=0.005) and less misclassifications as neutral (p=0.003) by rrMDD vs. controls. For neutral faces there was a significant (mis-)classification*group interaction (p=0.005). *p <0.05; ** p<0.01; rrMDD, remitted recurrent depressive disorder.

We excluded 1 rrMDD-patient who did not complete the task. Remitted rMDD-patients showed no differences in reaction times to any type of emotion (Table 2; independent *t*-tests, all *p*'s > 0.21; Figure 4). However, as shown in Figure 5, rrMDD-patients showed an increased recognition of sad faces, and more often misclassified stimuli as angry and disgusting (independent *t*-tests; [*t*_(108)_ = 2.01; *p* = 0.047], [*t*_(108)_ = 2.14; *p* = 0.035] and [*t*_(108)_ = 1.98; *p* = 0.050], respectively). Relative to controls, rrMDD-patients recognized neutral faces less well (independent *t*-test; [*t*_(108)_ = 2.49; *p* = 0.014], while they misclassified emotional faces less often as neutral (independent *t*-test; [*t*_(108)_ = 2.96; *p* = 0.004]. Only for neutral faces there was a significant (mis-)classification^*^group interaction (repeated measures ANOVA; [*F*_(1,108)_ = 8.33; η^2^ = 0.07; *p* = 0.005]. These findings did not change when we corrected for differences in HDRS between groups.

Next, we combined angry, fear, sad and disgusting expressions as negative faces, and surprised and happy as positive faces. When examining reaction times to positive or negative faces in a linear mixed model, we found no differences between rrMDD-patients and controls (main effect of group; *p* = 0.595), and in both groups a significant faster responses to positive than negative faces (main effect of valence; linear mixed model; [*F*_(1,428.00)_ = 75.98; *p* < 0.001]) without a valence^*^group interaction (*p* = 0.526; Figure 4). Comparisons of neutral with positive or negative faces, only showed significant slower reaction-times for negative and positive, relative to neutral faces ([*F*_(1,173.83)_ = 63.56; *p* < 0.001] and [*F*_(1,174.41)_ = 4.03; *p* = 0.046], respectively), without a significant main group effect or valence^*^group interaction (*p* > 0.248; Figure 4).

**Figure 6:**
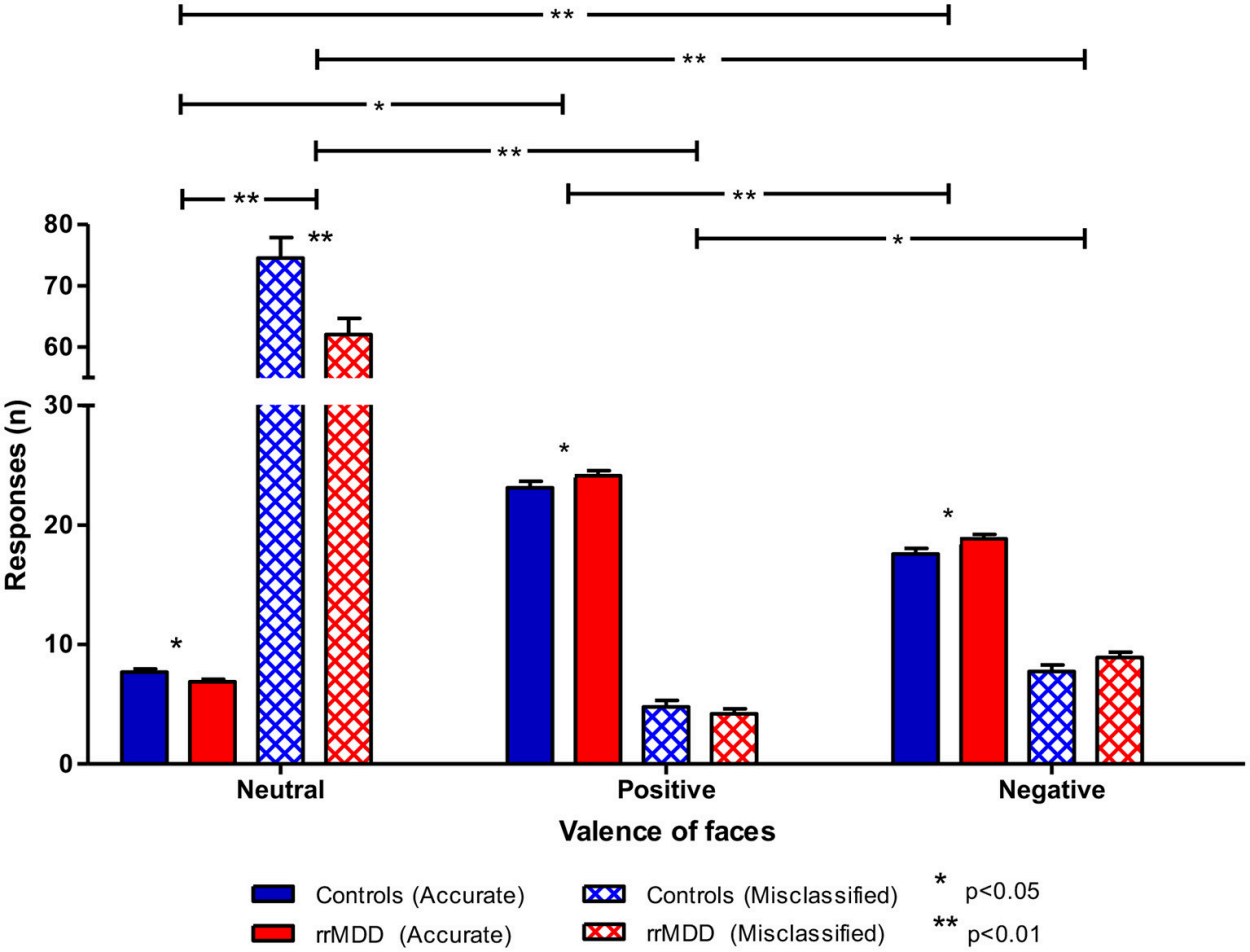
Accuracy of recognition of neutral vs. positive/negative facial expressions by rrMDD-patients vs. controls for the facial expression recognition task. Figure shows the number of emotional expressions (neutral vs. positive vs. negative) accurately recognized and misclassified as the indicated emotion for rrMDD-patients and controls. For accurate responses, for positive vs. negative faces we found significant main effects for group (*p* = 0.011), for positive or negative vs. neutral, there was a significant main effect for valence (better classification of positive or negative than neutral faces; *p* < 0.001), with a valence*group interaction [positive (*p* = 0.017); negative (*p* = 0.002)]. For misclassifications, for positive vs. negative faces we found significant main effects for valence (*p* = 0.011); for negative vs. neutral, there was a significant main effect for group (*p* = 0.009), valence (*p* < 0.001) and the valence*group interaction (*p* = 0.002); for positive vs. neutral, there was a main effect for group (*p* = 0.003), valence (*p* < 0.001) and the valence*group interaction (*p* = 0.006).* *p* < 0.05; ** *p* < 0.01; rrMDD, remitted recurrent depressive disorder.

For accuracy, using the same categorization, for positive vs. negative faces we observed better accuracy in rrMDD than in controls (main effect of group; linear mixed model; [*F*_(1,471.71)_ = 6.45; *p* = 0.011]), better accuracy for positive vs. negative faces (main effect of valence; [*F*_(1,471.71)_ = 144.47; *p* < 0.001]) without a valence^*^group interaction (*p* = 0.765; Figure 6). For the accuracy of classifications as positive or negative vs. neutral, in general, positive or negative faces were better classified than neutral (main effect of valence; linear mixed model; all *p*'s < 0.001), with a significant valence^*^group interaction (worse classification of neutral faces by rrMDD and better classification of positive and negative faces by rrMDD; [*F*_(1,264.55)_ = 5.82; *p* = 0.017] and [*F*_(1,533.90)_ = 9.54; *p* = 0.002], respectively).

For misclassifications of facial expressions, all subjects misclassified faces more often as negative than positive (main effect of valence; linear mixed model; [*F*_(1,346.85)_ = 60.31; *p* < 0.001]), without a significant difference between groups or valence^*^group interaction (both *p*'s > 0.077). For neutral vs. negative faces, we observed less misclassifications by rrMDD (main effect of group; [*F*_(1,113.91)_ = 7.01; *p* = 0.009]), more misclassifications as neutral (main effect of valence; [*F*_(1,113.91)_ = 786.75; *p* < 0.001) which was driven by rrMDD-patients having less misclassifications as neutral and more misclassifications as negative (significant valence^*^group interaction; [*F*_(1,113.91)_ = 10.23; *p* = 0.002]). Likewise, for positive vs. neutral, we observed significantly less misclassifications by rrMDD (main effect of group; [*F*_(1,114.08)_ = 9.35; *p* = 0.003]), more misclassifications as neutral (main effect of valence; [*F*_(1,114.08)_ = 890.14; *p* < 0.001]) again driven by less misclassifications as neutral in rrMDD but with a comparable number of misclassifications as positive between rrMDD-patients and controls (significant valence^*^group interaction; [*F*_(1,114.08)_ = 7.76; *p* = 0.006]; Figure 6).

### Follow-Up and Associations With Recurrence

Of the 64 MDD-patients who had at least 1 follow-up measurement, 35 (54.7%) had a recurrence, within a median period of 233 days (IQR 92-461). Patients with a recurrence had a younger age of onset (Independent *T*-Test; *p* = 0.035), more previous episodes in the last 10 years (Mann-Whitney; *p* = 0.001) but did not differ with respect to residual symptoms (*p* = 0.85; Table 3).

**Table 3:** Baseline characteristics of rrMDD patients recurrent vs. resilient during follow-up.

				**Between-group statistics**			
		**Recurrence (*n* = 35)**	**Resilient (*n* = 29)**	**χ^**2**^**	***T***	***U***	***p***
Female	*N* (%)	25 (71.4%)	19 (65.5%)	0.26			0.61
Age	Years; mean (SD)	52.8 (7.1)	54.7 (8.6)		0.98		0.37
Education	Levels^a^	0/0/0/2/15/13/5	0/0/0/2/6/12/9	4.52			0.21
IQ	Mean (SD)	108.0 (7.5)	110.0 (9.5)		0.90		0.37
Living situation	Levels^b^	15/0/9/7/2/0/2	11/0/9/9/0/0/0	4.34			0.40
Employment status	Levels^c^	15/15/5/0	9/10/10/0	3.64			0.16
Currently employed	Yes (%)	22 (64.7)	20 (71.4)	0.32			0.60
Age of onset	Years; mean (SD)	24.2 (10.7)	30.1 (11.0)		2.16		0.04
Episodes	Median (IQR)						
last 10 years lifetime		2 (1–3) 4 (2–12.5)	1 (1–2) 5 (2–5.5)			746 606	0.01 0.18
HDRS	Median (IQR)	2 (1–5)	2 (0–4.5)			521	0.85
Childhood adversity (CTQ)	Mean (SD)						
Total Emot. abuse Phys. abuse Sex. abuse Emot. neglect Phys. neglect		52.0 (15.5) 12.1 (5.8) 7.0 (2.9) 6.9 (3.2) 17.0 (5.5) 8.9 (3.4)	45.6 (11.5) 10.3 (4.8) 5.4 (1.2) 6.7 (3.1) 14.7 (4.9) 8.3 (3.1)		1.78 1.33 2.93 0.36 1.76 0.74		0.08 0.19 0.005 0.72 0.08 0.47

*Legend see Table 1*.

In the comparison of baseline results of rrMDD-patients without vs. those with a recurrence, we used significant comparisons and interactions with controls from Table 2 to calculate outcome-specific composite scores. Patients with a recurrence during follow-up significantly more often misclassified faces as angry than resilient patients (Mann-Whitney; *p* = 0.037), all other comparisons were not significant (*p* > 0.17; Table 4).

**Table 4:** Baseline emotional biases (expressed as outcome-specific composite scores) in rrMDD patients recurrent vs. resilient during follow-up.

				**Between-group statistics**		
		**Recurrent** **(*n* = 35)**	**Resilient** **(*n* = 29)**	***T***	***U***	***p***
**Emotional Categorization (EmCat)**						
Positive Mis. + Negative Mis.	*n* (SEM)	3.8 (0.65)	4.4 (0.68)		445	0.39
RT Neg. – RT Pos. Acc.	ms (SEM)	114.5 (27.45)	92.4 (23.22)		609	0.17
RT Neg. – RT Pos. Mis.^*^	ms (SEM)	−87.5 (173.17)	−198.9 (179.22)	−0.45		0.66
**Emotional Memory (EmMem)**						
Negative Acc. – Positive Acc.	n (SEM)	−0.14 (0.36)	0.00 (0.32	0.29		0.78
Negative Mis. – Positive Mis.	n (SEM)	−1.20 (0.35)	−0.79 (0.43)		518.5	0.69
Positive Acc. – Positive Mis.	n (SEM)	1.09 (0.56)	1.29 (0.29)	0.32		0.75
Negative Acc. – Negative Mis.	n (SEM)	2.14 (0.38)	2.07 (0.47)		471.5	0.80
(Negative Mis. – Positive Mis.)/(Positive Acc. – Negative Acc)^§^	% (SEM)	−54.5 (41.2)	−4.6 (44.8)		250.5	0.25
**Facial Emotion Recognition (FERT)**						
Sad Acc.	n (SEM)	16.7 (1.13)	16.9 (1.22)	0.89		0.93
Neutral Acc.	n (SEM)	6.5 (0.34)	7.2 (0.36)	1.41		0.69
Angry Mis.	n (SEM)	16.1 (1.85)	11.1 (1.46)		662	**0.04**
Disgust Mis.	n (SEM)	7.0 (1.08)	6.6 (0.98)		513.5	0.94
Neutral Mis.	n (SEM)	60.6 (3.59)	62.9 (3.89)	0.45		0.66
Positive Acc. – Neutral Acc.	n (SEM)	40.3 (1.25)	38.8 (1.88)	−0.65		0.52
Negative Acc. – Neutral Acc.	n (SEM)	67.4 (2.85)	67.7 (3.33)	0.06		0.95
Negative Mis. – Neutral Mis.	n (SEM)	−17.9 (6.06)	−28.9 (5.60)	−1.31		0.19
Neutral Mis. – Positive Mis.	n (SEM)	41.7 (3.78)	45.0 (4.57)	0.55		0.58

*Outcome-specific composite scores were defined based on significant differences and interactions of outcomes between patients and controls (see Table 2)*.

* *As not all subjects misclassified characteristics during emotional categorization, these mean reaction-times are based on less subjects (16 with recurrence and 16 resilient)*.

§ *Cases with Positive Acc. – Negative Acc = 0 omitted from analyses (7 recurrent /7 resilient)*.

*Bold value indicate significance at p < 0.05*.

**Table 5:** Cox proportional hazards models.

	**Unit**	**Exp(B)**	**95% CI**	***p* (Wald)**	***p* (model)**
**Emotional Categorization (EmCat)**					
*Previous episodes−10 years*	*n*	*1.119*	*1.042–1.203*	*0.002*	
*HDRS-score residual symptoms*	*1 point*	*1.014*	*0.880–1.168*	*0.849*	*0.003*
Positive Mis. + Negative Mis.	n	0.970	0.871–1.080	0.567	
RT Neg. – RT Pos. Acc.	10 ms	1.013	0.986–1.041	0.345	
RT Neg. – RT Pos. Mis.^*^	10 ms	1.003	0.995–1.011	0.423	
**Emotional Memory (EmMem)**					
*Previous episodes−10 years*	*n*	*1.118*	*1.040–1.201*	*0.002*	
*HDRS-score residual symptoms*	*1 point*	*1.019*	*0.886–1.171*	*0.797*	*0.004*
Negative Acc. – Positive Acc.	n	0.923	0.748–1.138	0.453	
Negative Mis. – Positive Mis.	n	0.918	0.744–1.132	0.422	
Positive Acc. – Positive Mis.	n	0.924	0.763–1.120	0.422	
Negative Acc. – Negative Mis.	n	0.981	0.848–1.135	0.798	
(Negative Mis. – Positive Mis.)/(Positive Acc. – Negative Acc)^§^	%	0.934	0.755–1.155	0.528	
**Facial Emotion Recognition (FERT)**					
*Previous episodes−10 years*	*n*	*1.117*	*1.038–1.202*	*0.003*	
*HDRS-score residual symptoms*	*1 point*	*0.983*	*0.848–1.139*	*0.818*	
*Angry Mis*.	*n*	*1.038*	*1.006–1.070*	*0.019*	*0.001*
Sad Acc.	n	1.044	0.948–1.148	0.382	
Neutral Acc.	n	0.923	0.660–1.290	0.637	
Disgust Mis.	n	0.968	0.887–1.057	0.467	
Neutral Mis.	n	1.062	0.895–1.261	0.490	
Positive Acc. – Neutral Acc.	n	1.019	0.950–1.092	0.603	
Negative Acc. – Neutral Acc.	n	0.996	0.937–1.060	0.905	
Negative Mis. – Neutral Mis.	n	1.028	0.966–1.095	0.383	
Neutral Mis. – Positive Mis.	n	0.992	0.924–1.065	0.819	

*Outcome-specific composite scores were defined based on significant differences and interactions of outcomes between patients and controls (see Table 2). Variables in italics represent the final models, for which a p-value (χ^2^) is given. All models contained previous episodes (last 10 years) and HDRS-score (residual symptoms). Selection of additional variables was done by forward stepwise selection from all listed outcome variables for each emotional bias task separately*.

* *As not all subjects misclassified characteristics during emotional categorization, these mean reaction-times are based on less subjects (16 with recurrence and 16 resilient)*.

§ *Cases with Positive Acc. – Negative Acc = 0 omitted from analyses (7 recurrent/7 resilient)*.

Second, examining associations with recurrence in Cox-proportional hazard models (all correcting for residual symptoms and previous episodes in the last 10 years), we found that only the misclassification of faces as angry in the FERT was significantly associated with time to recurrence (Wald = 5.52; *p* = 0.019). Of the a priori defined task-based composite scores only the standardized residuals of the EmMem was significantly associated with time to recurrence (Wald = 4.21; *p* = 0.040). The planned combinations of task-based composite scores were not significantly associated with recurrence.

#### Support Vector Machine Classifiers to Predict Recurrence

The accuracies and sensitivity/specificity of different classifiers are displayed in Table 6. In the table we show how different combinations of neuropsychological tasks and demographic information (number of previous episodes in last 10 years, residual symptomatology, age and gender, also extended with CTQ-scores) perform when different percentages of available features are selected. The best classifier had a significantly better accuracy of 78.1% relative to the *a-priori* recurrence rate in the sample of this study (54.7%) (EmCAT + EmMem + demographic/CTQ data; 10% features; *z* = 2.8; 1-sided *p* = 0.002). However, when inspecting the 4 predicting parameters in this SVM-outcome, these were only demographic/CTQ-items (number of previous episodes in last 10 years, age of onset, CTQ-physical abuse subscale-score and CTQ-physical abuse ≥8). Moreover, when running the SVM on the extended demographic predictor set only, a 50% features solution (containing age, number of previous episodes in last 10 years, age of onset, CTQ-emotional abuse, CTQ-physical abuse, CTQ-emotional neglect subscale-scores, CTQ-total score, CTQ-physical abuse ≥8 and CTQ-emotional neglect ≥15) provided approximately the same predictive accuracy (75.0%; *z* = 2.4; 1-sided *p* = 0.008). The best model containing neuropsychological features approximating this result was the FERT + demographics/CTQ (10% features) classifier (containing number of previous episodes in last 10 years, age of onset, CTQ-physical abuse subscale-score, FERT misclassifications as angry and FERT misclassifications as negative; 70.3%; *z* = 1.8; 1-sided *p* = 0.034).

**Table 6:** Performance of different Support Vector Machine algorithms predicting recurrence.

		**Percentage of features selected**		
		**10%**	**50%**	**100%**
**Info in algorithm**	**Total features**	**Accuracy (Sens/Spec)**	**Accuracy (Sens/Spec)**	**Accuracy (Sens/Spec)**
EmCat + EmMem	22	50.0 (54.3/44.8)	35.9 (48.6/20.7)	34.4 (34.3/34.5)
FERT	31	46.9 (42.9/51.7)	35.9 (34.3/37.9)	59.4 (65.7/51.7)
EmCat + EmMem + demographics	26	57.8 (60.0/55.2)	54.7 (57.1/51.7)	45.3 (40.0/51.7)
EmCat + EmMem + demographics (extended)	39	78.1 (68.6/89.7)^*^ [2.8071; 0.002]	56.3 (54.3/58.6)	45.3 (31.4/62.1)
FERT + demographics	35	54.7 (45.7/65.5)	60.9 (62.9/58.6)	54.7 (57.1/51.7)
FERT + demographics (extended)	48	70.3 (60.0/82.8)^*^ [1.8257; 0.034]	64.1 (68.6/58.6)	59.4 (60.0/58.6)
EmCat + EmMem + FERT	53	34.4 (28.6/41.4)	40.6 (45.7/34.5)	34.4 (34.3/34.5)
EmCat + EmMem + FERT + demographics	57	50.0 (40.0/62.1)	48.4 (51.4/44.8)	42.2 (45.7/37.9)
EmCat + EmMem + FERT + demographics (extended)	70	67.2 (57.1/79.3)	48.4 (60.0/34.5)	45.3 (42.9/48.3)
Demographics (extended) only	17	64.1 (51.4/79.3)	75.0 (71.4/79.3)^*^ [2.4066; 0.008]	56.3 (54.3/58.6)

*The SVM models were validated using a leave one out procedure (see methods). Data were used from all 64 participants for whom follow-up was available. Missing data points (e.g., for choices which were not made by a particular participant) were imputed as the mean of the training set. The C parameter was estimated over 50 values from 0.01 to 100 (the value used for prediction was the one which produced the highest accuracy in the training set). Demographics included gender, age, number of episodes in last 10 years and residual symptoms (HDRS-score), if indicated extended with CTQ-data*.

The classifiers marked with

* *performed significantly better than the a-priori recurrence-rate in the current sample (54.7%), between [] z-score and 1-tailed p-value are given. Positive and negative predictive values were not displayed as these are dependent on the recurrence-rate in the present sample*.

*EmCat, Emotional Categorization Task; EmMem, Emotional Memory; FERT, Facial Emotion Recognition Task; Sens, Sensitivity; Spec, Specificity*.

## Discussion

We assessed biased processing of emotional material in different cognitive domains (i.e., self-referent, emotional memory and interpretation biases) in a drug-free remitted recurrently depressed sample. We found that rrMDD-patients show biases toward emotionally negative stimuli (i.e., faster responses to negative self-relevant characteristics, better recognition of sad faces, worse recognition of neutral faces with more misclassifications as angry or disgusting faces and less misclassifications as neutral faces), of which the number of misclassifications as angry and the overall performance in the emotional memory task were also associated with the time to recurrence during 2.5 years of follow-up. In data-driven SVM classifiers, especially demographic and childhood adversity parameters, but also combined with misclassifications as angry/negative faces showed significant better prediction of recurrence-status. Overall, our data suggests persisting emotional biases when patients with recurrent depression are in remission, which are -at least partly- prospectively associated with recurrence.

Negative biases have been repeatedly observed in acutely depressed individuals, while findings in remitted or high-risk groups have been mixed (7, 76). Moreover, the associations with new episodes have been investigated less (49, 76), and only for self-referent biases. Below we will discuss our findings for different aspects of the biases we investigated in this study.

### Bias in Self-Relevant Material (EmCAT)

With the mixed model analyses of the emotional categorization task, enabling the investigation of combinations of positive/negative characteristics and accuracy, we found a bias in self-relevant information processing: first, rrMDD-patients generally made more mistakes in adequately recognizing positive or negative characteristics than controls; second, in line with our hypothesis, relative to controls, rrMDD-patients were faster in response to negative characteristics and slower in response to positive characteristics, while especially for incorrect responses to positive characteristics this difference was the largest. However, contrary to our hypothesis, the reaction times to negative or positive characteristics separately or in combination (mean reaction-time negative–mean reaction time positive) were not associated with recurrence-risk over 2.5 years.

It has been proposed by earlier research, that a lack of a protective positivity bias observed in depressed individuals might be another component of depression existing independently from a negativity bias (76). In contrast to depressed individuals, euthymic healthy individuals appeared to have a positive attentional bias, in contrast to depressed individuals, who may often lack such a “protective” bias (14). Since we investigated euthymic subjects who were previously depressed, our valence^*^group^*^accuracy interaction is indicative of both increases in negative and decrease of positive self-relevant bias in rrMDD-patients, which is different from controls (i.e., rrMDD-patients have a negative bias and lack a protective bias). Nevertheless, in the current sample, the difference between reaction times to accurately identified negative and positive characteristics was not associated with recurrence.

Negative biases in self-referent material have been found in remitted MDD patients vs. controls before, e.g., when using the SRET (77). In a recent study by LeMoult et al. euthymic female individuals with a history of depression exhibited negatively biased self-referential processing (less positive and more negative words endorsed) during the SRET, however assessed *after* a negative mood induction (49). The latent SRET variable (additionally including memory of negative words) was found to prospectively predict episode recurrence over 3 years of follow-up (49).

Methodological differences might explain the discrepancy between our and these findings. First, the use of a mood induction in this study might have increased the negative biases in participants, in line with the cognitive reactivity model, and may have probed the vulnerability for recurrence. This would imply that self-referent biases might be latent in remission and mood-congruent only, instead of persistently present independent of mood-state. If so, we might have observed a negative bias if we would have applied a mood-induction before the EmCAT. Second the difference in SRET vs. the EMCAT task (explicitly referring to oneself vs. valence of characteristics in relation to oneself; i.e., self-referent vs. self-relevant/partly self-referent) might have influenced the variability of correct responses, since most subjects determined the right valence for most characteristics in the EmCAT. This might have reduced the possibility to find associations with recurrence and EmCAT outcomes. Next, the approach of summarizing the outcomes of the SRET, including the memory in one latent SRET-measure as predictor of recurrence (49) might also explain the different findings since combination of information might increase sensitivity to detect biases. Finally, in our non-mood-induced EmCAT, we found most robust interactions regarding reaction times, which could not be modeled by LeMoult et al. (49). Again, although we did not find associations with recurrence, differences in reaction times might have been more sensitive to predict recurrence when obtained after a mood-induction (expected to increase the differences in reaction times between positive and negative adjectives).

### Bias in Memory of Emotional Material (EmMem)

In contrast with our hypotheses, bias in emotional memory was not different between rrMDD-patients and controls. In the emotional memory task, we only found better recall of positive words, with more words remembered correctly than incorrectly and a significant accuracy^*^valence interaction (no difference between positive and negative words when recalled correctly, but more positive than negative words when recalled incorrectly). Interestingly, despite the absence of significant differences between rrMDD-patients and controls on separate outcome variables, the task-based composite score (indicating the individual's deviation of the general pattern of differences between patients and controls) was associated with recurrence.

In previous studies, recall of negative words was increased in rrMDD in investigations with the SRET (49, 77), which was accompanied by unexpected recall afterwards. In addition Vrijsen et al. also reported increased negative memory bias for negative stimuli in remitted MDD after a sad mood induction, which was not specifically associated with having recurrent MDD (48). Interestingly, Gethin and colleagues reported that reductions in positivity bias in a comparable sample of remitted MDD-patients were only found in subjects reporting early life stress (47). In *post-hoc* analyses, approximating the analyses by Gethin et al. we did not find evidence for an effect of early life stress [assessed by the CTQ (71, 72)] on reductions of recall of positive (relative to negative) words in our sample (results available on request). As noted above, LeMoult et al. reported an association with recurrence of the SRET-results, containing a variable for memory of negative words (49). Given the fact that our task-based composite score is relative to the present control sample, the association with prospective recurrence is interesting but will need replication and preferably must be substituted by an absolute value independent of a control sample.

The mood induction before, and the shorter time between the SRET and recall (3 min) (49) compared to this study (no mood-induction; time between ECAT and recall 15 min) might both be relevant factors that might have reduced variability between subjects in our study; these in turn might have obscured associations between memory bias and recurrence. Moreover, it has been suggested that the level of self-reference of the presented characteristics and/or the overgeneralization of autobiographical memories (i.e., reduced ability to recall specific autobiographical memories) are more important in the inability of rrMDD subjects to be resilient against recurrence (76, 78). Unfortunately, we did not test autobiographical memories in addition to the EmCAT/EmMem.

### Bias in Recognition of Faces (FERT)

In the facial expression recognition task, contrary to our hypotheses for reaction times, there were no overall or valence -specific differences in reaction times between groups. However, in line with our hypothesis of bias toward negatively valenced faces, rrMDD-patients better recognized sad faces, more often misclassified stimuli as angry and disgusting and exhibited poorer recognition of neutral faces than controls. Further, they misclassified emotional faces less often as neutral. Moreover, in interaction analyses, rrMDD-patients showed worse classification of neutral faces and better classification of positive and negative faces. This was complemented by less misclassifications as neutral but more misclassifications as negative (and comparable misclassifications as positive) faces by rrMDD-patients vs. controls. Of these findings, only the increased misclassification of faces as angry was significantly associated with time to recurrence during 2.5 years of follow-up. This finding was corroborated by the SVM classifier that included the FERT-outcomes and revealed a significant classification with 50% of the features.

Depressed patients show mood-congruent biases in the identification of facial expressions of emotion (76, 79, 80). In line with our findings, earlier research described that these biases in the identification of facial expressions of emotion appear to remain after recovery from a depressive episode (41, 45, 81). Joorman et al. (45) showed that formerly depressed participants selectively attended sad faces, while controls selectively avoided sad faces and oriented toward happy faces instead, indicative of a positive bias that was not observed in remitted MDD-patients. Leppanen et al. (41) used neutral, happy and sad faces only, and found in their analyses of remitted MDD-patients vs. controls that these patients misclassified neutral faces more often (and equally) as either sad or happy, while we found more misclassifications (from either valence) as angry in rMDD, but -comparably- identified worse recognition of neutral faces by rrMDD. LeMoult et al. (81) also used a different task (with computer-morphed variable intensity of emotions) while also including a mood induction procedure: they observed differences in recognition of happy emotions while we found an increased recognition of sad and more misclassification as angry faces. Unfortunately, LeMoult et al. did not report the misclassifications as angry and neither of these two studies performed a follow-up to associate biases with recurrence (41, 81).

We expect that our and Leppanen et al.'s non-mood-induced results point to a trait-like difficulty in recognizing neutral expressions, presumably as they see them as more negative, while the mood-induction used by LeMoult might have elicited mood-congruent (state-like) recognition/interpretation biases (41, 81). The finding that misclassifications were significantly more often toward angry faces could be hypothesized as representation of implicit expectations/anxiety of having done something wrong, i.e., self-blame as proposed by Zahn et al. (82), who reported that 80% of patients with remitted MDD report self-blaming feelings as a significant symptom in their last episode. This might persist as residual symptom/bias contributing to a general vulnerability for recurrence, according to the revised learned helplessness model in which subjects blame themselves for failure in an overgeneralized way (83). The relevance of this misrecognition of neutral stimuli as negative, might be that a difficulty in accurately identifying subtle expression of emotion will hinder effective interpersonal interactions and/or social support in daily life (76). Since individuals use facial expressions to monitor emotional reactions to determine others' opinions and to adjust their behavior (76), important for social interactions, we propose that -in line with the general risk for depression of such impairments (84)- this impairment also plays an important role in recurrence. In fact, the observed association of recurrence with increased misclassifications as angry corroborates this idea. Moreover, the observed worse recognition of happy information/stimuli/faces when in a dysphoric mood (81) and the proposed difficulties in the processing of positive affect in MDD in general (76, 79–81) might additionally decrease resilience against (an impeding) recurrence. However, our facial recognition data suggest that the biases for positive material might be mood-congruent only, while difficulty in recognizing neutral expressions also exists without attempts to induce sad mood and are therefore “mood-incongruent” and might represent a trait (41).

### Strengths and Limitations

An important strength of this study is our prospective design with 2.5 years follow-up and ADM-free patient sample. Moreover, cross-sectional studies comparing patients and controls usually do not control for a multitude of confounding factors such as mood state, anxiety disorder co-morbidity and trauma which make interpretations more difficult. Pharmacological interventions might alter neuropsychological and specifically emotional information processing, which can be observed already hours after intake (85–87). By excluding (remitted) patients using antidepressants, we avoid any influence of antidepressants on emotional bias, which was not possible in earlier studies [e.g., (40, 49, 86, 88)]. Although selection of unmedicated rrMDD-patients might represent a less severe spectrum of the disease, the 55% recurrence rate rather contradicts this potential selection bias.

Nevertheless, some limitations must be addressed. First, as mentioned earlier, we did not apply a mood induction before measuring the cognitive biases reported in this manuscript. Previous research found that cognitive biases are present after recovery from a depressive episode but may remain dormant until activated by negative mood or stress (18). A mood-induction procedure may be required to reveal such biases. Although we deliberately performed the mood-induction procedure after these neurocognitive tasks (64), this might have obscured biases in tasks using self-relevant material (EmCAT, EmMem), as discussed. As euthymia does not exclude dysphoria or dysthymic affect, these fluctuations might have influenced the assessments, challenging their mood-incongruency. Nevertheless, we assessed severity of depression of all subjects when doing the tests and excluded patients who were depressed at the time of testing. Therefore, in absence of a mood-induction, we think we can interpret our results to represent more trait-like disturbances instead of sad mood congruent (i.e., state-dependent) phenomena. This might be relevant for daily life and clinical applicability where a mood-induction most often is unfeasible (89).

Second, emotional biases are more profound when stimuli are self-referent. The EmCAT must be considered partly self-referent (i.e., self-relevant), since we asked participants to indicate agreeableness of self-referent characteristics. It would be interesting to know whether the use of (verbal) self-referential material in e.g., a SRET or a memory task for autobiographical material would yield comparable differences between rrMDD-patients and controls and/or more associations with prospective recurrence. In addition, our assessment of emotional memory might be more sensitive by assessing retrieval in interaction with emotional load (90). Nevertheless, the validity of the tasks used and their sensitivity to detect biases has been shown previously, albeit primarily in depressed subjects (60, 61, 85).

Third, sex differences in emotion identification (e.g., in faces) have been identified in previous studies (81, 91, 92), therefore several studies included only women (49, 81). We included both sexes, which might have obscured our findings. *Post-hoc* analyses in the current study indeed revealed a gender^*^valence interaction for the accuracy of positive vs. neutral faces (FERT), but without a gender^*^valence^*^group interaction, which was our primary interest. However, for the significant accuracy^*^valence^*^group interaction for reaction times in the EmCAT we also found an interaction with gender (mixed model; accuracy^*^valence^*^group^*^gender interaction; [*F*_(5,3283.92)_ = 18.81; *p* < 0.001]). This indicated that male rrMDD-patients were both faster in response to positive (especially incorrect) and negative characteristics than male controls, while females rrMDD-patients were overall slower in response to both positive (especially incorrect) and negative characteristics (data available on request). This gender effect in the EmCAT needs further exploration in future studies.

Fourth, the number of observations of incorrect classifications of self-relevant characteristics in the EmCAT was low, which might therefore be a false-positive result, so this result should be considered preliminary. Also, the statistical power to observe associations with recurrence might have been too limited to exclude the possibility of false negative findings. Moreover, we did not apply a multiple comparison correction, so our results must be regarded as exploratory. Ideally, selecting variables for prospective prediction on the basis of their abnormality compared with healthy controls would also requiring multiple testing correction. When we would e.g., apply a Bonferroni correction, known to be the most conservative, the association with recurrence will be non-significant, which merits cautious interpretation of this result. Moreover, although SVM algorithms are widely used and robust, the leave-one-out cross validation method has been criticized for overestimating accuracy of prediction and poor generalization.

Fifth, the vulnerability to have a recurrence mediated by emotional biases might only become relevant in interaction with daily stressors or maybe more importantly: daily hassles (93). As such, such stressors/daily hassles might better be modeled as time-dependent covariates in future analyses.

Finally, Hertel concluded that depressed individuals have the ability to perform at the level of healthy control participants in structured situations but have difficulty doing so when situations are unconstrained or when they are left to their own initiative (94). Although we abstained from an artificial mood-induction when examining biases, our tests were also acquired in a laboratory setting, which might have reduced their sensitivity or generalizability (76).

## Conclusion

When investigating emotional biases in drug-free, remitted recurrently depressed patients, we observed biases toward emotionally negative stimuli and poorer recognition of neutral facial expressions. Overall, our data suggests a persisting (also mood-incongruent) emotional bias when patients with recurrent depression are in remission. Moreover, the number of misclassifications as angry-faces and the task-based composite score for the emotional memory were independently associated with the time to recurrence during 2.5 years of follow-up. We propose that these persisting biases might be mechanistically important for recurrence and prevention thereof.

## Author Contributions

HR, CH, and AS designed the study. HR, RM, and CF performed recruitment and data-acquisition of participants and daily management of the study. PS, RM, MB, and HR performed analyses and interpreted the data. HR drafted the manuscript with assistance of PS, RM, NI, AT, MB, and JV. All authors provided feedback on the initial versions of the manuscript and approved the final version.

### Conflict of Interest Statement

MB has received travel expenses from Lundbeck for attending conferences, and acted as a consultant for Johnson and Johnson. He is employed part time by P1vital Ltd and owns shares in P1vital Products Ltd. CH has served as a paid consultant or speaker for Lundbeck, Servier, P1vital, and Pfizer. She has received grant income from Johnson and Johnson, UCB, and P1vital. The remaining authors declare that the research was conducted in the absence of any commercial or financial relationships that could be construed as a potential conflict of interest.
